# Oxygen delivery index during goal-directed therapy predicts complications and hospital length of stay in patients undergoing high-risk surgery

**DOI:** 10.1186/cc11081

**Published:** 2012-03-20

**Authors:** M Cecconi, N Arulkumaran, R Suleman, D Shearn, M Geisen, J Mellinghoff, D Dawson, J Ball, M Hamilton, M Grounds, A Rhodes

**Affiliations:** 1 St George's Hospital, London, UK

## Introduction

The aim of this study was to evaluate the efficacy of a goal-directed therapy (GDT) protocol designed to augment the oxygen delivery index (DO_2_I) and to assess the relationship between DO_2_I measurements and postoperative complications and length of stay.

## Methods

A single-centre retrospective cohort study assessing the data obtained during an 8-hour post-operative GDT protocol in consecutive major surgical patients admitted to the ICU.

## Results

**Figure 1:**
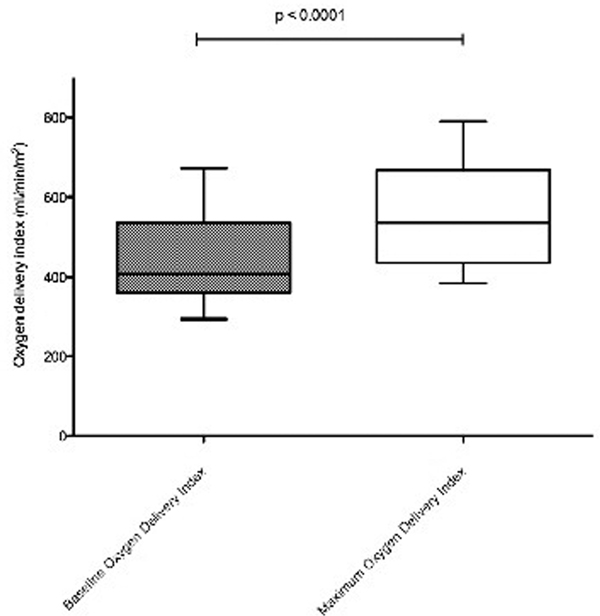
**Increase in DO_2_I from baseline to maximum over the 8-hour protocol**.

Thirty-seven patients were included. The median DO_2_I increased over the 8-hour protocol from a baseline level of 407 ml/minute/m^2 ^to a maximum of 537 ml/minute/m^2 ^(*P *< 0.0001) (Figure 1). Twenty-one (57%) patients developed a postoperative complication. Patients who developed zero or one complication had a higher maximum oxygen delivery index DO_2_I than patients who had more than one complication (602 vs. 477 ml/minute/m^2^, *P *= 0.018) (Table 1). The proportion of patients with a length of stay greater than 2 weeks was less in patients who achieved a DO_2_I of at least 600 ml/minute/m^2 ^(*P *= 0.035).

**Table 1:** Postoperative complications by achievement of an oxygen delivery index of 600

	DO_2_I >600	DO_2_I <600	*P *value
Number of patients	16 (43%)	21 (57%)	-
Complications	13 (29%)	32 (71%)	*P *= 0.003
Mortality	0 (0%)	4 (100%)	*P *= 0.12

## Conclusion

Postoperative GDT was able to increase DO_2_I in the postoperative period. Patients who achieved a DO_2_I of 600 ml/minute/m^2 ^were less likely to suffer postoperative complications and have a significantly reduced length of hospital stay.

